# Inhibition of the long non-coding RNA MALAT1 downregulates MAP2K1, suppressing the progression of hypopharyngeal squamous cell carcinoma

**DOI:** 10.17305/bb.2024.11151

**Published:** 2024-09-23

**Authors:** Xiaomin Wang, Hui Li, Aoxuan Xu, Jie Peng, Yanqing Wu, Yunfan Liu, Junjie Zhang, Changqi Cai, Shiyin Ma, Kai Zhang

**Affiliations:** 1 Anhui Medical University, Hefei, China; 2 Department of Otolaryngology and Head and Neck surgery, The First Affiliated Hospital of Bengbu Medical College, Bengbu, China; 3 Department of Oral and Maxillofacial Surgery, The First Affiliated Hospital of Bengbu Medical University, Bengbu, Anhui, China

**Keywords:** lncRNA, MALAT1, MAP2K1, hypopharyngeal squamous cell carcinoma, progression

## Abstract

This study aimed to explore the role of long non-coding RNAs metastasis-associated lung adenocarcinoma transcript (lncRNA MALAT1), and its underlying mechanisms in hypopharyngeal squamous cell carcinoma (HSCC). Quantitative real-time PCR (qRT-PCR) was employed to measure lncRNA MALAT1 expression in HSCC and adjacent non-cancerous tissues, along with the expression of the downstream target mitogen-activated protein kinase kinase 1 (MAP2K1). The independent prognostic significance of lncRNA MALAT1 was assessed using Cox regression analysis. Potential downstream targets of MALAT1 were identified through parallel reaction monitoring (PRM) analysis and validated using the TCGA-HNSC database, Western blotting, and immunohistochemistry. CCK-8, flow cytometry, and Transwell assays were conducted to assess the effects of the lncRNA MALAT1/MAP2K1 axis on FaDu cells. Additionally, a nude mouse xenograft model was used to confirm the role of lncRNA MALAT1/MAP2K1 in tumor growth. LncRNA MALAT1 was significantly upregulated in HSCC tissues and closely associated with poor prognosis. Bioinformatics analysis, including PRM screening and TCGA-HNSC data, identified FERMT2, CSNK2A2, and MAP2K1 as potential downstream targets of MALAT1. Validation through qPCR, Western blotting, and immunohistochemistry confirmed MAP2K1 as a downstream target. *In vitro* and *in vivo* experiments demonstrated that inhibiting lncRNA MALAT1 suppressed cell proliferation, migration, and epithelial-to-mesenchymal transition (EMT) by downregulating MAP2K1 expression. Additionally, it induced apoptosis, affected the cell cycle, and inhibited tumor growth. Our study uniquely demonstrates that targeting MALAT1 significantly impedes HSCC progression by downregulating its novel downstream target, MAP2K1, offering new insights into potential therapeutic strategies.

## Introduction

Hypopharyngeal squamous cell carcinoma (HSCC) is one of the most aggressive head and neck malignancies, with five-year survival rates ranging from 30% to 50% [1, 2]. Early-stage HSCC is often asymptomatic due to its hidden location, leading to frequent metastasis to cervical lymph nodes [3]. Standard treatments—surgery, radiotherapy, and chemotherapy—offer limited survival benefits, particularly for patients with intermediate and advanced stages [4, 5]. In recent years, immunotherapy and targeted therapy have emerged as promising options for patients with advanced recurrence and metastasis, although their applicability remains limited [6]. Accumulating evidence implicates multiple genes and functional RNAs in the complex molecular regulation of HSCC [7]. Further understanding of the mechanisms driving HSCC initiation, progression, and immune evasion is essential for identifying reliable prognostic biomarkers and improving therapeutic strategies.

Long non-coding RNAs (lncRNAs), which are RNA molecules over 200 nucleotides in length, play significant roles in cellular biology [8]. Among them, lncRNA metastasis-associated lung adenocarcinoma transcript (MALAT1) has been implicated in the pathogenesis of various cancers [9], including esophageal [10], cervical [11], and laryngeal cancers [12], where it influences tumor cell proliferation, apoptosis, invasion, and metastasis [13]. However, conflicting findings regarding its underlying mechanisms have been reported in the literature [14]. Previous investigations by our group identified upregulated expression of lncRNA MALAT1 in laryngeal squamous cell carcinoma tissues, closely correlating with patient prognosis [15]. Research on lncRNA MALAT1 in HSCC, however, remains limited, with few mechanistic insights. Understanding its role in HSCC could be clinically significant, offering potential diagnostic markers and therapeutic targets.

Mitogen-activated protein kinase kinase 1 (MAP2K1), a member of the dual-specificity protein kinase family, plays a critical role in transducing intracellular and extracellular signals within the Ras/Raf/MEK/ERK pathway, significantly influencing tumorigenesis and progression in various cancers. Notable examples include primary glioma [16], atypical BRAF-mutated melanoma [17], and non-small cell lung cancer [18]. In HSCC, activation of the mitogen-activated protein kinase (MAPK) pathway by factors, such as the CD147 ligand cyclophilin A, ETS transcription factors, and indoleamine 2,3-dioxygenase 1 (IDO1), has been implicated in promoting proliferation and metastasis [19]. The involvement of lncRNA MALAT1 in MAP2K1 activation and its potential regulatory role in HSCC proliferation and metastasis remain unclear. Therefore, this study employed quantitative real-time PCR (qRT-PCR) and Western blotting techniques to elucidate the relationship between lncRNA MALAT1 and MAP2K1 expression. Stable cell lines with silenced MALAT1 expression (shMALAT1) were established to investigate the impact of MALAT1 on the biological behavior of HSCC. Subsequently, *in vitro* cell experiments and *in vivo* animal models were used to demonstrate that the lncRNA MALAT1/MAP2K1 signaling pathway may influence HSCC progression by modulating epithelial–mesenchymal transition (EMT).

## Materials and methods

### Tissue samples

All HSCC tissue samples and patient information were collected following the same procedures as in our previous study [20]. The study adhered to the Declaration of Helsinki and was approved by the Research Ethics Committee of The First Affiliated Hospital of Bengbu Medical College. Written informed consent was obtained from all patients. The TNM stage was determined according to the 7th edition of the American Joint Committee on Cancer (AJCC) grading system.

### Cell culture and transfection

Human HSCC FaDu cells were obtained from the Cell Bank of the Chinese Academy of Sciences, Shanghai Institutes for Biological Sciences. The cells were cultured in DMEM/F12 medium supplemented with 10% FBS, 100 U/mL penicillin, and 100 U/mL streptomycin (Gibco, USA). Incubation was carried out in a 5% CO_2_ humidified incubator at 37 ^∘^C.

For transient transfection, X-TREMEGENE HP DNA Transfection Reagent (#06366236001, Roche) was used according to the manufacturer’s instructions. Stable transfected cell lines were generated using lentiviral transfection. Lentiviral packaging was provided by Biomedical (BG0000052GJ), with the lncMALAT1-sh vector: pLKO.1-EGFP-Puro. The vector sequence included U6-MCS-CMV-EGFP-hPGK-puro. The MAP2K1 overexpression plasmid vector (GENE ID: NM_002755.4) was pCDH-CMV-MCS-EF1-CopGFP-T2A-Puro. The lncMALAT1 interference target (Gene ID: 378938) was GAGTAACTGGCATGTGAGCAA (5′-3′). The designed interference sequence was: shlncMALAT1: GAGTAACTGGCATGTGAGCAACTCGAGTTGCTCACATGCCAGTTACTCTTTTT.

### Cell proliferation and migration assay

For the cell proliferation assay, FaDu cells were plated in a 96-well plate at a density of 2×10^3^ cells/well one day prior to the experiment. After cell adherence, 10 µL of CCK-8 solution (BIOMEDICAL, Austria) was added to the cells and incubated in the dark for 2 h. Absorbance was measured using a microplate reader at 450 nm.

For the migration assay, transfected cells were maintained in a serum-free medium and added to the top chambers of a transwell insert. The complete medium was added to the bottom chambers. After 24 h, the migrated cells were fixed with paraformaldehyde, stained with 0.1% crystal violet, and counted under a microscope.

### Detection of apoptosis and cell cycle

FaDu cells were transfected for 24 h and then centrifuged at 1300 rpm for 5 min. Next, 200 µL of the cell suspension was incubated with 2-µL Annexin V-FITC and 2-µL propidium iodide (PI), following the manufacturer’s protocol, in a dark environment at 4 ^∘^C for 30 min. Cell apoptosis analysis was performed using flow cytometry. For the cell cycle assay, FaDu cells were collected 24 h after transfection. The cells were stained with 1 mL of Cell Staining Solution (40× PI: 100× RNase: 1× D-Hanks ═ 25:10:1000) for 30 min in the dark. Flow cytometry was then used to detect cell cycle arrest.

### qRT-PCR assay

Tissue samples were immediately stored in liquid nitrogen after being obtained from patients during surgery to prevent RNA degradation. The samples were ground into fine particles using a tissue homogenizer and transferred into tubes containing lysis buffer. After gentle mixing and incubation, RNA was extracted from the lysate using Trizol reagent (Invitrogen). Reverse transcription (RT) of RNA was performed using an RT-PCR kit (TAKARA, Japan), according to the manufacturer’s instructions. For qRT-PCR analysis, cDNA was amplified using a StepOnePlus Real-Time PCR System (ABI, USA). The qRT-PCR was conducted using SYBR Green Master Mix (Applied Biosystems) under the following cycling conditions: initial denaturation at 95 ^∘^C for 10 min, followed by 40 cycles of 95 ^∘^C for 15 s and 60 ^∘^C for 60 s. The primers for lncRNA MALAT1 were: forward 5′-TCAAGGTAACGATGGTGTCGA-3′ and reverse 5′-CCACTCAAATGCCTATCTTCTC-3′. The primers for MAP2K1 were: forward 5′-CCTTGAGGCCTTTCTTACCC-3′ and reverse 5′-CCCACGATGTACGGAGAGTT-3′. The primers for GAPDH were: forward 5′-AGAAGGCTGGGGCTCATTTG-3′, and reverse 5′-AGGGGCCATCCACAGTCTTC-3′. GAPDH amplification was used as an internal control.

### RNA scope in situ assay

The RNA Scope Multiplex Fluorescent Reagent kit (ACD, USA) was used to examine the expression of lncRNA MALAT1 in HSCC tissues and adjacent tissues, following the manufacturer’s instructions.

### Tandem mass tag (TMT)

After protein extraction, quantification, SDS-PAGE electrophoresis, and staining of each group of samples, an appropriate amount of protein solution was enzymatically digested using the FASP method, lyophilized through C18 cartridge desalination, redissolved with formic acid solution, and quantified. The FASP digestion peptide segment was labeled according to the instructions of Thermo’s TMT labeling kit, then mixed and graded. Quantitative proteomic analysis using TMT was performed on a QExactivePlus mass spectrometer after separation by the EasynLC system. Mascot 2.6 and Proteome Discoverer 2.2 software were used to search the database for identification and quantitative analysis. The Uniprot_HomoSapiens_20367_20200226 database was selected, and reliable qualitative results were obtained by screening data according to FDR<0.01 standard.

### Parallel reaction monitoring (PRM)

After sample protein extraction, SDS-PAGE electrophoresis, and FASP enzymatic digestion as described in section 2.2, peptides of equal quality from each sample were mixed, separated by Ultimate 3000 chromatography, and analyzed by an online electrospray tandem QExactivePlus mass spectrometer. Proteome Discoverer 2.1 software was used to convert the original chromatogram files generated by the mass spectrometer into.mgf files. The MASCOT 2.6 server was used for database retrieval, using the Uniprot_HomoSapiens_20367_20200226 database. The unique peptide of the target protein was filtered to obtain information about its mass–charge ratio, charge number, and retention time, which was then added to the inclusion list.

### Bioinformatics analysis

Proteins with an expression difference of more than 1.2-fold and a *P* value (*t*-test) less than 0.05 were considered differentially expressed proteins. A volcano plot was generated for a significant difference analysis of the target proteins. Gene Ontology (GO) annotation was performed using Blast2GO, and KEGG pathway annotation was conducted using KOALA software. In the enrichment analysis of GO annotation or KEGG pathway annotation, the significance of protein enrichment for a specific GO term or KEGG pathway was evaluated using Fisher’s exact test. matplotlib software was used to classify the expression levels of both samples and proteins, generating hierarchical clustering heat maps. Based on the TCGA-HNSC database (https://portal.gdc.cancer.gov/), gene expression analysis and prognosis analysis were performed using R language (v3.60), with differential analysis conducted using the “limma” package and prognostic analysis using the “survival” package.

### Western blotting

Proteins were extracted from lentivirus-infected FaDu cells using an EDTA-free pancreatic enzyme and a PMSF-containing lysis buffer, and the protein concentration was determined using a BCA reagent. After incubation, the optical density at a wavelength of 562 nm was measured with an enzyme-linked analyzer under light-avoiding conditions. The separation gel was prepared according to standard protocols, and SDS-PAGE gel electrophoresis was performed after loading the samples. The proteins were transferred to a PVDF membrane using Tris-Glycine transfer buffer, followed by washing and blocking as per the sealer’s instructions. Western blotting was conducted using specific primary antibodies, including anti-MAP2K1 (1:1000 dilution) and anti-GAPDH (1:2000 dilution), with overnight incubation at 4 ^∘^C. Membranes were then exposed to secondary antibodies (1:2000 dilution) for 1 h at room temperature, followed by detection using ECL substrate (Thermo Scientific), with exposure times adjusted depending on signal strength (typically 5 min). Immunohistochemistry was performed using anti-MAP2K1 (1:1000 dilution) and diaminobenzidine (DAB) as the chromogen, with a 10-min incubation. The SuperSignal^®^ West Dura kit was used for signal development, and ImageJ software analyzed the strip optical density values, with each strip repeated three times. Results were expressed as mean ± standard deviation.

### *In vivo* tumor growth assay

To assess the role of the MALAT1/MAP2K1 pathway in tumor growth, we conducted an *in vivo* tumor growth assay using a nude mouse xenograft model. Male BALB/c (nu/nu) nude mice were purchased from Shanghai Lingchang Biotechnology Co., Ltd. and housed in the SPF animal laboratory. A total of 30 male BALB/c (nu/nu) nude mice were randomly assigned to three groups (ten mice per group). FaDu cells (5 × 10^7^ cells/mL) infected with shMALAT1, shMALAT1 + MAP2K1-OE, or negative control lentivirus were implanted into the mice (200 µL per mouse). Tumor growth was monitored every three days using digital calipers, and tumor volume was calculated using the formula: tumor volume (mm^3^) ═ (width^2^ × length)/2, where width and length represent the shortest and longest tumor dimensions, respectively. The study continued for 36 days, after which the mice were sacrificed, and tumors were harvested for further analysis. All animal experiments were conducted in accordance with Institutional Animal Care and Use Committee (IACUC) guidelines to ensure ethical treatment of animals.

### Immunohistochemistry

Paraffin sections were placed in dewaxing solutions I, II, III, anhydrous ethanol, alcohol, and distilled water for rehydration. After antigen retrieval, the sections were preincubated with 10% goat serum to block nonspecific binding. HSCC tissue was used as a positive control. Primary antibodies were incubated overnight at 4 ^∘^C, including anti-MAP2K1 (abcam, ab32091, 1/5000 dilution, Rabbit), Anti-Ki67 (abcam, ab15580, 1/500 dilution, Rabbit), Anti-E-Cadherin (abcam, ab314063, 1/1000 dilution, Rabbit), and Anti-N-cadherin (abcam, ab76011, 1/5000 dilution, Rabbit), Anti-Vimentin (abcam, ab92547, 1/1000 dilution, Rabbit). After primary antibody incubation, sections were incubated with a biotinylated secondary antibody (Vector Labs) for 1 h at room temperature, followed by incubation with the ABC reagent (Vector Labs) for 30 min. Peroxidase activity was visualized using a DAB kit (Vector Labs), resulting in a brown color. Slides were counterstained with hematoxylin (Sigma) to visualize the nuclei. The stained sections were then examined and images were captured using a microscope camera (Zeiss, Germany).

### Ethical statement

All animal experiments were conducted in accordance with the Nursing and Use Guidance for Animal Experiment Operations from the National Institutes of Health (NIH Pub. no. 85-23, revised in 1996) and were approved by the Ethics Committee of The First Affiliated Hospital of Bengbu Medical University (Approval number: 2022409).

### Statistical analysis

Data were analyzed using SPSS 22.0 statistical software. Measurements following or approximating a normal distribution are expressed as mean ± standard deviation. Independent sample *t*-tests were used for comparisons between two groups, while one-way analysis of variance was used for comparisons among multiple groups, and *P* < 0.05 was considered statistically significant. All experiments, including animal studies, were performed with at least three independent replicates to ensure reproducibility. Multivariate Cox regression analysis was used to evaluate the independent prognostic significance of lncRNA MALAT1. Variables included in this analysis were age, gender, smoking status, alcohol consumption, tumor stage (T stage), nodal stage (N stage), and lncRNA MALAT1 expression levels, and *P* < 0.05 was considered statistically significant.

## Results

### Expression and prognosis value of lncRNA MALAT1 in hypopharyngeal carcinoma

**Figure 1.**
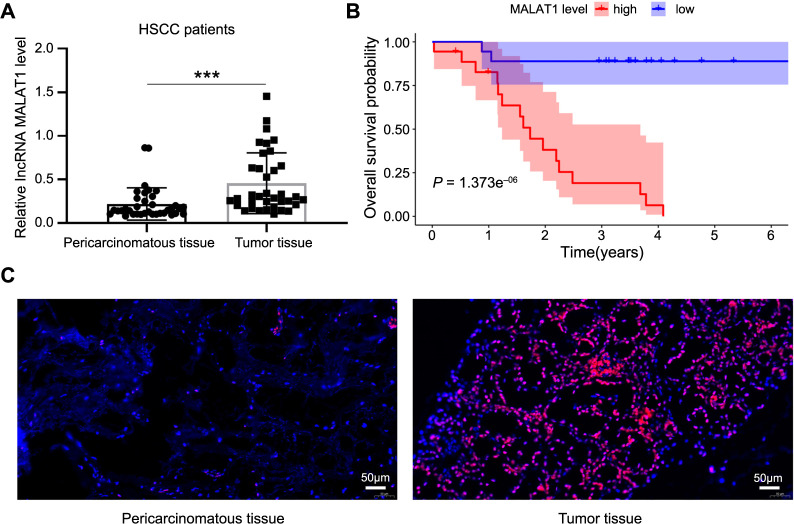
**Expression and prognosis value of lncRNA MALAT1 in hypopharyngeal carcinoma.** (A) lncRNA MALAT1 expression is significantly elevated in 36 HSCC patients compared to adjacent non-cancerous tissues; (B) Kaplan–Meier survival analysis showing overall survival (OS) rates stratified by high and low lncRNA MALAT1 expression levels, based on data from the TCGA database; (C) Representative RNA scope images illustrating lncRNA MALAT1 expression in matched HSCC and paracancerous tissues (*n* ═ 36). Scale bar ═ 50 µm. ***P < 0.001. lncRNA MALAT1: Long non-coding RNAs metastasis-associated lung adenocarcinoma transcript; HSCC: Hypopharyngeal squamous cell carcinoma.

To assess the expression of lncRNA MALAT1 in patients with HSCC, we collected 36 pairs of surgical tissue samples (Table 1). In this table, smoking was found to significantly correlate with lncRNA MALAT1 expression. However, this correlation did not remain significant in the multivariate Cox survival analysis, suggesting that while smoking may influence MALAT1 expression, it is not an independent prognostic factor for HSCC survival. This highlights the complexity of factors influencing prognosis in HSCC and underscores the importance of considering multiple variables in survival analysis. In the multivariate Cox regression analysis, key variables, such as age, gender, smoking status, alcohol consumption, tumor stage (T stage), nodal stage (N stage), and lncRNA MALAT1 expression levels were included. Both univariate and multivariate analyses indicated that lncRNA MALAT1 is a high-risk factor for hypopharyngeal cancer survival (Tables 2 and 3). Furthermore, we found that lncRNA MALAT1 was significantly overexpressed in hypopharyngeal cancer tissues (Figure 1A), and patients with higher MALAT1 expression had a significantly worse prognosis (Figure 1B). Consistent with the RT-PCR results, RNAscope also showed that lncRNA MALAT1 was significantly overexpressed in hypopharyngeal cancer tissues. It was primarily localized in subnuclear plaques (highly dynamic subnuclear structures) and may regulate variable splicing by promoting the shuttle transport of SR proteins between nuclear plaques and gene transcription sites (Figure 1C).

**Table 1:** Clinical features of 36 HSCC patients

**Clinicopathologic parameters**	**lncRNA MALAT1 expression** **Low (*n* ═ 18)**		**LncRNA MALAT1 expression** **High (*n* ═ 18)**		**χ^2^**	***P* value**
Age					0.111	0.739
< 60	9		8			
> ═ 60	9		10			
Gender					1.029	0.311
Female	0		1			
Male	18		17			
Smoker					4.300	0.038
No	3		8			
Yes	15		8			
Alcohol history					2.786	0.095
No	7		12			
Yes	11		6			
T stage					0	1
T1 + T2	6		6			
T3 + T4	12		12			
N stage					0	1
N0	6		6			
N1-N3	12		12			
M stage					0	1
M0	18		18			
M1	0		0			
Clinical stage					0	1
Stage I + Stage II	1		1			
Stage III + Stage IV	17		17			
OS event					21.778	<0.0001
Alive	16		2			
Dead	2		16			

HSCC: Hypopharyngeal squamous cell carcinoma; lncRNA MALAT1: Long non-coding RNAs metastasis-associated lung adenocarcinoma transcript.

**Table 2:** Univariate Cox regression of overall survival and clinicopathologic characteristics in 36 HSCC patients

**Clinicopathologic parameters**	**Univariate cox regression**			
	**HR**	**HR. L (95% CI)**	**HR.H (95% CI)**	***P* value**
Gender	0.497	0.065	3.806	0.501
Age	0.983	0.930	1.038	0.528
Smoke	0.980	0.949	1.012	0.215
Alcohol	1.000	0.998	1.002	0.969
Stage	1.355	0.620	2.962	0.447
T	0.992	0.544	1.812	0.980
N	1.204	0.697	2.079	0.505
lncRNA MALAT1	46.988	9.779	225.782	<0.0001

HSCC: Hypopharyngeal squamous cell carcinoma; lncRNA MALAT1: Long non-coding RNAs metastasis-associated lung adenocarcinoma transcript; T: Tumor stage (T stage); N: Nodal stage (N stage).

**Table 3:** Multivariate analyses of overall survival and clinicopathologic characteristics in 36 HSCC patients

**Clinicopathologic parameters**	**Multivariate analyses**			
	**HR**	**HR. L (95% CI)**	**HR.H (95% CI)**	***P* value**
LncRNA MALAT1	94.008	11.676	756.858	<0.0001

HSCC: Hypopharyngeal squamous cell carcinoma; lncRNA MALAT1: Long non-coding RNAs metastasis-associated lung adenocarcinoma transcript.

### Knockdown of lncRNA MALAT1 inhibited the malignant biological behavior of FaDu cells *in vitro*

**Figure 2.**
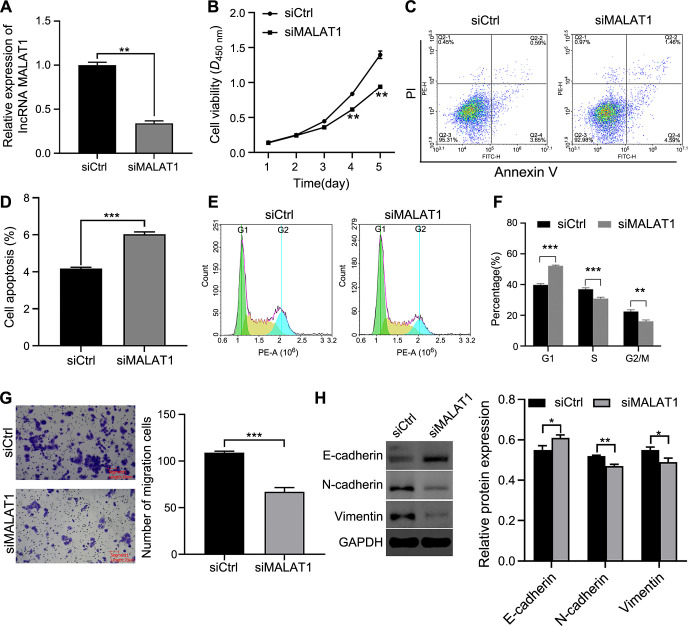
**Knockdown of lncRNA MALAT1 inhibited malignant biological behavior of FaDu cells.** (A) qRT-PCR detection of MALAT1 expression after transfection of FaDu cells with siRNA inhibiting MALAT1; (B) CCK-8 assay showing reduced proliferation of FaDu cells following MALAT1 knockdown; (C and D) Flow cytometry analysis of apoptosis in control and MALAT1-knockdown FaDu cells. Representative images (left) and quantitative analysis (right) are shown; (E and F) Flow cytometry analysis of cell cycle distribution in control and MALAT1-knockdown FaDu cells. Representative cell cycle profiles (left) and cell stage quantifications (right) are shown; (G) Transwell assay demonstrating decreased migration ability in MALAT1-knockdown FaDu cells compared to controls. Representative images (left) and quantifications (right) are shown; (H) Western blot analysis of E-cadherin, N-cadherin, and Vimentin protein levels in control and MALAT1-knockdown FaDu cells. Data are presented as mean ± SD from three independent experiments, *P < 0.05, **P < 0.01, ***P < 0.001. lncRNA MALAT1: Long non-coding RNAs metastasis-associated lung adenocarcinoma transcript.

To investigate the loss-of-function role of lncRNA MALAT1 in HSCC cell proliferation, apoptosis, cell cycle, migration, and EMT, lncRNA MALAT1 siRNA or siRNA negative control was transfected into FaDu cells to alter its expression. As shown in Figure 2A, the expression of lncRNA MALAT1 in the siMALAT1 group was significantly lower than in the siCtrl group (*P* < 0.01). The CCK-8 assay revealed that the knockdown of lncRNA MALAT1 significantly impaired FaDu cell proliferation (*P* < 0.01, Figure 2B). Flow cytometry analysis indicated that inhibition of lncRNA MALAT1 increased the apoptosis rate of FaDu cells (*P* < 0.001, Figure 2C and 2D). Likewise, the percentage of FaDu cells in the G1 phase significantly increased, while the percentage of cells in the S and G2/M phases decreased following lncRNA MALAT1 silencing (*P* < 0.001, Figure 2E and 2F). Additionally, the migration ability of FaDu cells was reduced in the siMALAT1 group compared with the siCtrl group (*P* < 0.001, Figure 2G). Western blot analysis showed increased protein expression of E-cadherin after lncRNA MALAT1 inhibition, while the protein levels of N-cadherin and Vimentin were reduced (*P* < 0.05, Figure 2H). These findings suggest that knockdown of lncRNA MALAT1 suppresses cell proliferation, migration, and EMT, while promoting cell cycle arrest and apoptosis in FaDu cells.

### Screening of differentially expressed proteins

**Figure 3.**
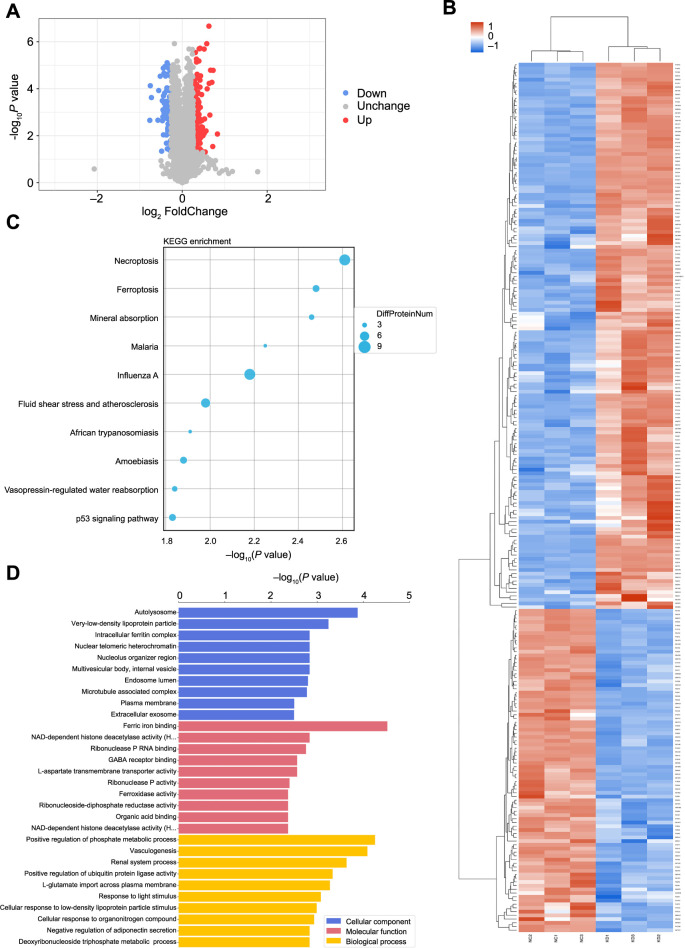
**Summary of proteomics analysis following MALAT1 knockdown.** (A) Volcano plot illustrating differentially expressed proteins between control and MALAT1-knockdown groups, highlighting proteins with significant upregulation and downregulation; (B) Heat maps showing protein clustering in the control and knockdown groups. The vertical axis represents the protein cluster, while the horizontal axis represents the sample cluster; (C) Bubble diagram of KEGG enrichment analysis for differentially expressed proteins following proteomic sequencing, with circle size indicating the number of differential proteins in each pathway; (D) GO enrichment analysis of differentially expressed proteins after proteomic sequencing. MALAT1: Metastasis-associated lung adenocarcinoma transcript; GO: Gene Ontology.

Proteomic sequencing identified a total of 6044 quantifiable proteins. Proteins meeting the criteria of an expression difference ratio greater than 1.2 (up- or downregulation) and a *P* value (*t*-test) less than 0.05 were considered differentially expressed. A total of 234 differential proteins were identified, with 147 upregulated and 87 downregulated (Table 4). Specific expression details can be seen in the volcano plot (Figure 3A).

**Table 4:** Statistics of protein quantitative results

**Comparisons**	**Up-**	**Down-**	**All-**
KD VS NC	147	87	234

Up-: Upregulated differentially expressed protein; Down-: Downregulated differentially expressed protein; All-: All differentially expressed proteins; KD: Knockdown of lncRNA MALAT1; NC: Negative control.

### Bioinformatics analysis of differentially expressed proteins

We performed cluster analysis to group the differentially expressed proteins in both the control and knockdown groups, and presented the data as heat maps (Figure 3B). Additionally, we identified proteins associated with the target gene “MAP2K1” from the STRING (version 12.0) database and mapped the protein–protein interaction network. The results showed strong interactions between MAP2K1 and proteins of the RAS family (KRAS, HRAS, NRAS) and the RAF family (BRAF, ARAF, RAF1), suggesting a critical role for MAP2K1 in the RAS-RAF-MEK-ERK signaling pathway (Figure S1).

GO functional annotation and enrichment analysis, KEGG pathway annotation and enrichment analysis, and cluster analysis were also performed. The results of KEGG pathway annotation and enrichment analysis are shown in Figure 3C. Based on *P* value rankings, necroptosis (nine differentially expressed proteins) was the pathway most significantly enriched with differentially expressed proteins. Other pathways included ferroptosis (five differentially expressed proteins), mineral absorption (four differentially expressed proteins), malaria (three differentially expressed proteins), and influenza A (nine differentially expressed proteins) (refer to Appendix 1).

GO is a standardized functional classification system. The GO annotation of the target protein collection includes biological process (BP), molecular function (MF), and cellular component (CC). As shown in Figure 3D, BPs were mainly concentrated in the positive regulation of phosphate metabolic processes (33 significantly differentially expressed proteins) and vasculogenesis. CCs were primarily enriched in autolysosomes (four differentially expressed proteins), very low-density lipoprotein particles, and intracellular ferritin complexes. MFs were mainly focused on ferric iron binding (four differentially expressed proteins) and NAD-dependent histone deacetylase activity, among others (refer to Appendix 2).

### Analysis of IPA-related words

PathwaysKnockdown of lncRNA MALAT1 has been reported to affect transcription and/or pre-mRNA splicing of key genes involved in migration and cell adhesion, as well as genes involved in cancer pathways related to invasion, metastasis, and chemotherapy resistance. The enrichment analysis of the previous TMT results revealed that differentially expressed proteins were significantly enriched in pathways related to necrotic apoptosis and ferroptosis. Metastasis, a key hallmark of cancer progression, involves numerous factors such as extracellular matrix (ECM) degradation, EMT, tumor angiogenesis, the development of an inflammatory tumor microenvironment, and programmed cell death. Programmed cell death, including necrotic apoptosis, autophagy, and ferroptosis, plays a crucial role in tumor metastasis. As one of the most malignant tumors in the head and neck, HSCC is characterized by aggressive invasion, frequent lymph node involvement, and distant metastasis. Therefore, we used IPA analysis to further investigate PRM analysis of genes enriched in tumor phenotype-related pathways, including EMT, tumor invasion, metastasis, and DNA damage (refer to Appendix 3).

### PRM preliminarily analyzed the potential downstream regulatory molecules of MALAT1

**Figure 4.**
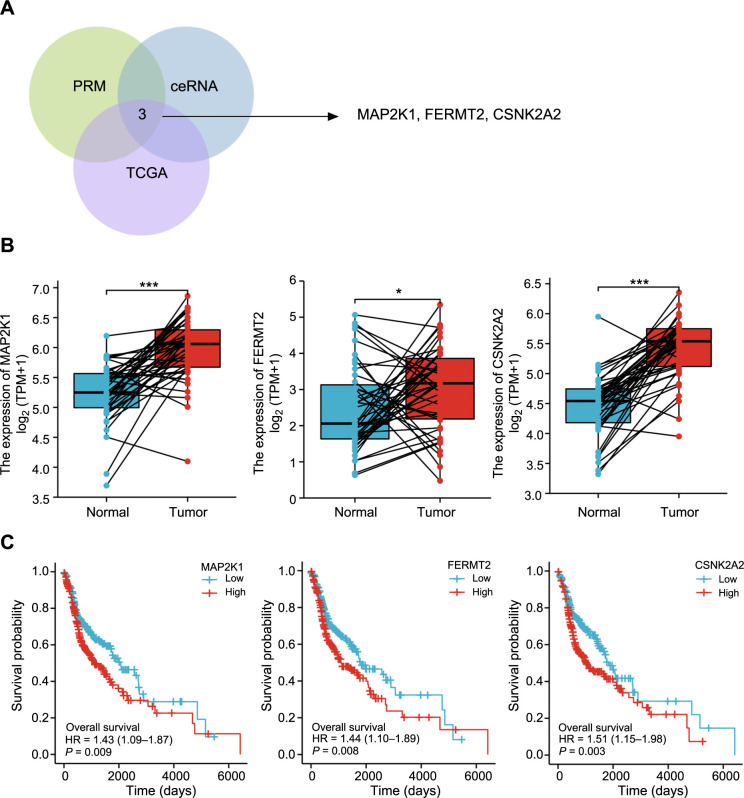
**Candidate biomarkers were validated by the TCGA dataset.** (A) Venn diagram of common sites predicted by PRM, ceRNA mechanism and TCGA-HNSC validation; (B) Differential expressions of MAP2K1; FERMT2 and CSNK2A2; (C) Kaplan–Meier curves of overall survival (OS) of MAP2K1, FERMT2 and CSNK2A2 in TCGA-HNSC cohort data. MAP2K1: Mitogen-activated protein kinase kinase 1; MAP2K1: Mitogen-activated protein kinase 2 kinase 1; FERMT2: Fermitin Family Member 2; CSNK2A2: Casein kinase 2 alpha 2 subunit.

The candidate peptides of 29 target proteins were analyzed by PRM (refer to Appendix 4). The quantitative results of PRM showed that 28 of the 29 candidate proteins corresponded well with the TMT data, and the trend consistency between PRM verification and TMT data was 96.55%, indicating the reliability of the data obtained by TMT (refer to Appendix 5). Quantitative analysis of PRM protein data showed that NIPSNAP2, HSPB1, CALB2, HMGB2, DHCR24, HBA1, FAF2, MAP2K1, FERMT2, STK26, and THRAP3 were significantly downregulated, while FTH1, F3, STMN1, KRT15, CSNK2A2, NUMB, RAB5C, ARHGDIB, CDK6, GLO1, STK3, GANAB, and GBF1 were significantly upregulated. Among these genes, seven proteins (MAP2K1, FERMT2, HMGB2, FAF2, STK26, THRAP3, and KRT15) showed significant differences between carcinoma and adjacent tissues (TCGA-HNSC database). Only MAP2K1 and FERMT2 had potential prognostic value (Figure 4A–4C). Through the ceRNA mechanism, 16 genes were identified at the intersection of all MALAT1 target genes (*n* ═ 9303, from this source, refer to Appendix 6). These include NIPSNAP2, HSPB1, DHCR24, FAF2, MAP2K1, FERMT2, THRAP3, FTH1, STMN1, CSNK2A2, NUMB, RAB5C, CDK6, STK3, GANAB, and GBF1, of which only CSNK2A2 had significant expression differences and prognostic value as validated by the TCGA-HNSC (Figure 4A–4C) (The expression of other proteins is presented in Figure S2).

### The protein expression of prognostic genes was verified by WB and immunohistochemistry

**Figure 5.**
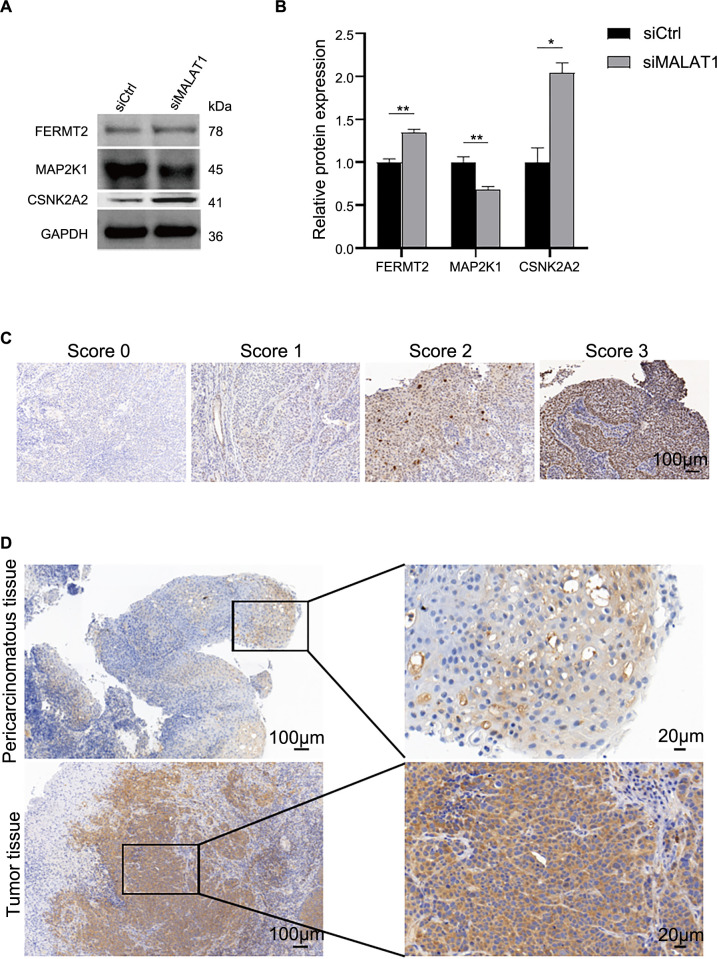
**Candidate biomarkers were validated by the Western blotting and immunohistochemistry.** (A and B) Western blotting detection of protein expression of FERMT2, CSNK2A2, and MAP2K1 after knockdown of MALAT1 in FaDu cells. And quantification of protein expression of FERMT2; CSNK2A2, and MAP2K1 after knockdown of MALAT1 in FaDu cells. Data indicate mean ± SD of triplicate samples. (*P < 0.05, **P < 0.01, ***P < 0.001); (C) IHC staining were divided into four grades according to the staining intensity: 0, 1, 2 and 3; (D) Immunohistochemistry was used to detect the expression of MAP2K1. Hematoxylin stained the nucleus blue, and the positive expression of DAB was brownish yellow. MALAT1: Metastasis-associated lung adenocarcinoma transcript; MAP2K1: Mitogen-activated protein kinase kinase 1; DAB: Diaminobenzidine; MAP2K1: Mitogen-activated protein kinase 2 kinase 1; FERMT2: Fermitin Family Member 2; CSNK2A2: Casein kinase 2 alpha 2 subunit.

We further verified the expression of differential prognostic genes (FERMT2, CSNK2A2, MAP2K1) in FaDu cells. The results of Western blotting (Figure 5A) showed that the expression levels of FERMT2 and CSNK2A2 proteins were significantly increased (*P* < 0.05). The expression of MAP2K1 protein was significantly decreased (*P* < 0.001). Quantitative results were confirmed through gray analysis (Figure 5B). We then performed immunohistochemical staining in cancer tissues and adjacent paracancerous tissues to detect MAP2K1 protein expression. Staining intensity was divided into four levels: 0, 1, 2, and 3, following the study by Zhu et al. [21] (Figure 5C). Immunohistochemical results (Figure 5D) showed high expression of MAP2K1 protein in cancer tissues, while adjacent tissues showed low or absent expression. These results suggest that MAP2K1 may be a downstream target of lncRNA MALAT1.

### LncRNA MALAT1 silencing suppressed malignant biological behavior of FaDu cells by downregulating MAP2K1

**Figure 6.**
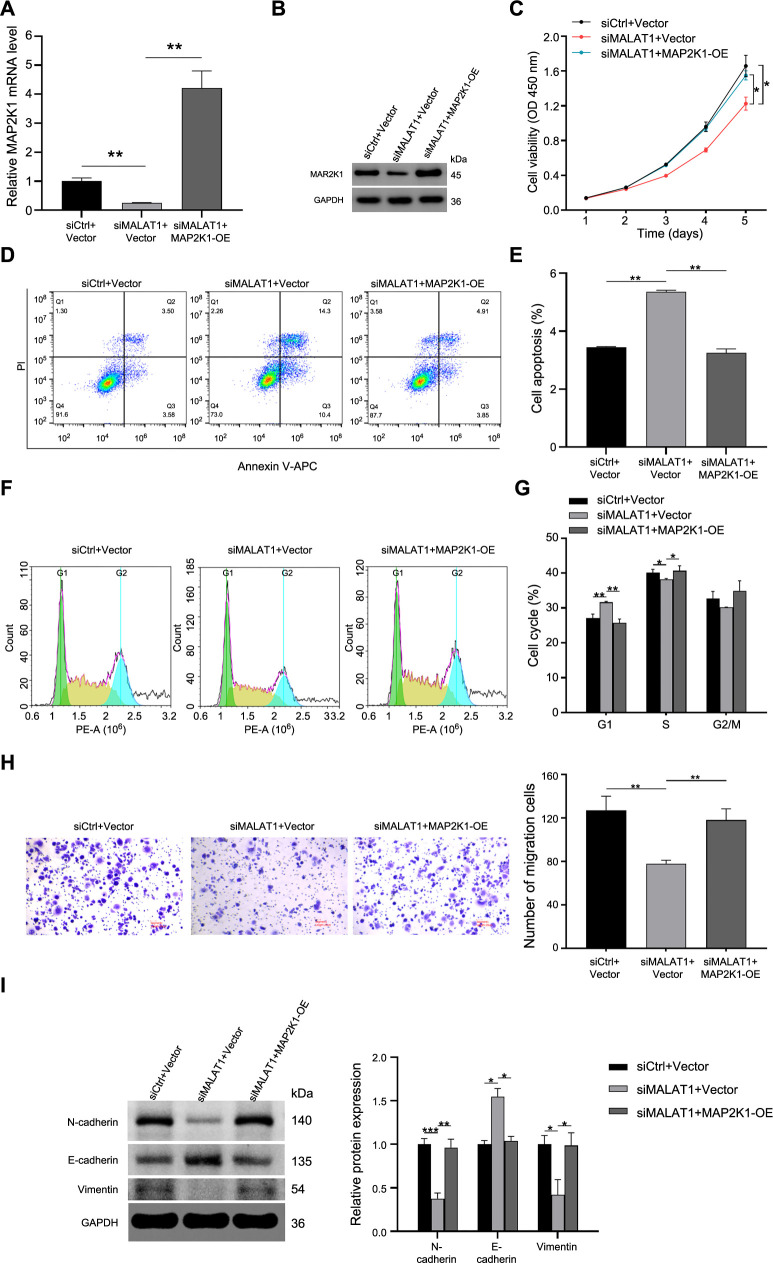
**Upregulation of MAP2K1 reversed the inhibitory effect of lncRNA MALAT1 silencing on the malignant biological behavior of FaDu cells.** (A) The mRNA expression of MAP2K1 in FaDu cells after MAP2K1 overexpression was detected by qRT-PCR; (B) Western blotting was applied to detect the protein expression of MAP2K1; (C) CCK-8 assay was performed to evaluate FaDu cell proliferation; (D and E) Flow cytometry assay was employed to measure FaDu cell apoptosis; (F and G) Flow cytometry assay was used to evaluate FaDu cell cycle distribution; (H) Transwell assay was used to test FaDu cell migration, Scale bar ═ 20 µm; (I) Western blotting was applied to detect the protein expression of E-cadherin, N-cadherin, and Vimentin. Protein levels were normalized to GAPDH. (*P < 0.05, **P < 0.01, ***P < 0.001). lncRNA MALAT1: Long non-coding RNAs metastasis-associated lung adenocarcinoma transcript; MAP2K1: Mitogen-activated protein kinase kinase 1.

To further assess whether lncRNA MALAT1 affects the proliferation, apoptosis, cell cycle, and migration abilities of FaDu cells by regulating MAP2K1 *in vitro*, we conducted a rescue assay. qRT-PCR showed that the mRNA expression of MAP2K1 was significantly increased after MAP2K1 overexpression via pcDNA-MAP2K1 in FaDu cells (*P* < 0.01, Figure 6A). Western blot analysis showed a decrease in MAP2K1 expression following MALAT1 knockout. Conversely, enforced expression of MAP2K1 after MALAT1 knockout resulted in an elevated expression of MAP2K1 (Figure 6B). Upregulation of MAP2K1 significantly reversed the inhibitory effect of lncRNA MALAT1 knockdown on FaDu cell proliferation (*P* < 0.05, Figure 6C). Moreover, the promotion of apoptosis (*P* < 0.001, Figure 6D and 6E) and cell cycle arrest (*P* < 0.05, Figure 6F and 6G) by lncRNA MALAT1 knockdown was significantly abrogated by MAP2K1 overexpression in FaDu cells. Furthermore, MAP2K1 overexpression markedly alleviated the inhibitory effects of lncRNA MALAT1 deficiency on FaDu cell migration (*P* < 0.01, Figure 6H). Compared to the siMALAT1 group, decreased protein expression of E-cadherin and increased protein expression of N-cadherin and Vimentin were observed in the siMALAT1+MAP2K1 group (*P* < 0.05, Figure 6I). These findings suggest that the knockdown of lncRNA MALAT1 exerts an anti-HSCC effect by downregulating MAP2K1.

**Figure 7.**
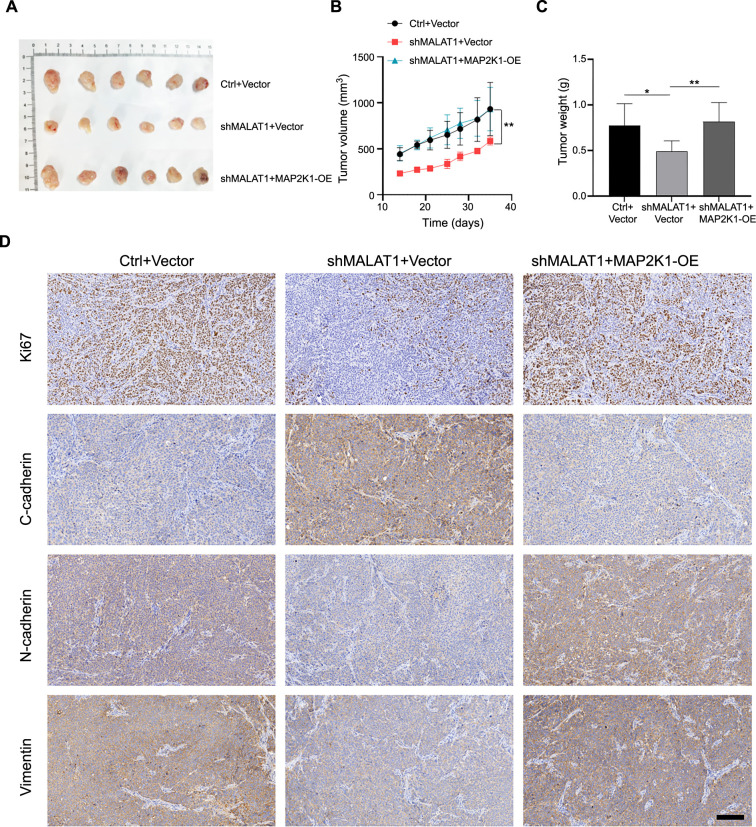
**Knockdown of lncRNA MALAT1 impeded tumor growth by reducing MAP2K1 expression.** (A) Representative images of tumors excised from the xenograft model; (B) Tumor volume growth curves for control and MALAT1-knockdown groups over time; (C) Measurement of final tumor weights after the experiment; (D) Immunohistochemical staining of tumor tissues for Ki-67, E-cadherin, and N-cadherin. Scale bar ═ 50 µm. (*P < 0.05, **P < 0.01, ***P < 0.001). lncRNA MALAT1: Long non-coding RNAs metastasis-associated lung adenocarcinoma transcript; MAP2K1: Mitogen-activated protein kinase kinase 1.

### Inhibition of lncRNA MALAT1 reduced tumor growth by downregulation of MAP2K1 *in vivo*

To further verify the functional role of the lncRNA MALAT1-MAP2K1 axis *in vivo*, a xenograft model was constructed. FaDu cells with stable lncRNA MALAT1 knockdown and/or MAP2K1 overexpression were implanted into nude mice. Knockdown of lncRNA MALAT1 significantly reduced tumor volume compared to the control group, while MAP2K1 overexpression produced the opposite effect (Figure 7A and 7B). Additionally, the tumor weight in the shMALAT1 group was markedly lower than that in the control group, whereas it was higher in the shMALAT1+MAP2K1-OE group (Figure 7C). Immunohistochemical staining revealed lower positive expression of Ki-67 and N-cadherin, and higher positive expression of E-cadherin, in the shMALAT1 group compared to the control group. However, the opposite results were observed in the shMALAT1+MAP2K1-OE group (Figure 7D). Collectively, lncRNA MALAT1 knockdown impeded tumor growth by reducing MAP2K1 expression.

## Discussion

In recent years, targeted therapy has emerged as a precise approach with significant advantages, leading to the identification of numerous therapeutic targets [6]. To impede the progression of HSCC, discovering additional therapeutic targets is imperative. Our current investigation revealed elevated expression levels of lncRNA MALAT1 in human HSCC tissues. Bioinformatics analysis identified several enriched pathways, such as necroptosis and ferroptosis, among differentially expressed proteins following MALAT1 knockdown. However, due to the scope and focus of this study, we did not perform functional studies to directly confirm the involvement of these pathways in the context of MALAT1 knockdown. This decision was made to prioritize the investigation of the MALAT1/MAP2K1 axis, representing a novel and significant finding in HSCC pathology. Nevertheless, these enriched pathways offer valuable insights and will be key targets for future research to further elucidate their roles in MALAT1-mediated tumor progression. Functionally, our findings demonstrate that increased MAP2K1 expression counteracts the inhibitory effects observed upon lncRNA MALAT1 knockdown, thereby promoting malignant biological behaviors and tumor growth in HSCC cells.

Numerous studies have established lncRNA MALAT1 as an oncogenic factor implicated in the pathogenesis and progression of various malignancies [22]. Many studies identify elevated expression of MALAT1 in malignant tumor tissues and cell lines [23, 24], and our study is consistent with these findings. Clinical investigations have further demonstrated that increased MALAT1 levels correlate with larger tumor size, lymph node metastasis, and poorer overall survival among cancer patients [25]. Additionally, MALAT1 has emerged as a promising diagnostic and therapeutic target across multiple cancer types [26]. Collectively, these observations underscore MALAT1’s potential as a robust biomarker for HSCC and other malignancies.

Recently, lncRNA MALAT1 has emerged as a key regulator of cancer cell biology, influencing diverse processes including proliferation, apoptosis, invasion, migration, and EMT [27]. Our study demonstrates that MALAT1 knockdown in FaDu cells attenuates proliferation, migration, and EMT while promoting apoptosis and inducing cell cycle arrest. Consistent with our findings, Zhu et al. [28] reported similar effects of MALAT1 depletion in tongue squamous cell carcinoma, highlighting its role in regulating cell proliferation, invasion, and migration. Accumulating evidence indicates that MALAT1 serves as a pivotal determinant in diagnosing metastatic potential across various malignancies [29], exhibiting notably elevated expression in recurrent colorectal cancer [30] and metastatic lesions [31]. Experimental studies further reveal that MALAT1 knockdown mitigates lung and lymph node metastasis in animal models [32]. Overall, these findings underscore MALAT1’s multifaceted role in cancer progression, suggesting its potential as a therapeutic target and biomarker for metastatic disease.

MAP2K1, a pivotal member of the MAPK family [33], plays a crucial role in regulating the malignant phenotypes of cancer cells, including proliferation, apoptosis, invasion, and migration. Previous investigations have established MAP2K1 as an oncogenic driver with aberrantly elevated expression in various malignancies [34]. In our study, elevated MAP2K1 expression was observed in HSCC tissues, and its levels were attenuated in FaDu cells following lncRNA MALAT1 knockdown. Notably, overexpression of MAP2K1 counteracted the anti-tumor effects induced by MALAT1 suppression in HSCC, promoting FaDu cell proliferation and invasion while diminishing apoptosis and hindering cell cycle arrest. Consistent with these findings, recent studies have underscored the role of MAP2K1 depletion in inhibiting the proliferation, migration, and invasion of cancer cells across diverse cancers, including hepatocellular carcinoma [35], gastric cancer [33], and esophageal squamous cell carcinoma [36]. These observations emphasize MAP2K1’s significance as a potential therapeutic target in cancer treatment strategies.

## Conclusion

In conclusion, one of the key findings of this study is the identification of MAP2K1 as a novel downstream target of lncRNA MALAT1 in HSCC. While the oncogenic role of MALAT1 has been previously documented in various cancers, the specific involvement of MAP2K1 in the MALAT1-mediated regulatory network within HSCC has not been reported until now. Our research uniquely demonstrates that downregulation of MAP2K1 is a critical mechanism by which MALAT1 inhibition suppresses tumor progression, including proliferation, migration, and EMT processes. This novel insight not only expands our understanding of the molecular underpinnings of HSCC but also positions MAP2K1 as a potential therapeutic target in MALAT1-driven cancers. By establishing the MALAT1-MAP2K1 axis as a significant contributor to HSCC pathology, our study opens new avenues for targeted therapeutic interventions aimed at disrupting this pathway to improve patient outcomes.

## Supplemental data

Supplementary data are available at the following links: https://www.bjbms.org/ojs/index.php/bjbms/article/view/11151/3541 https://www.bjbms.org/ojs/index.php/bjbms/article/view/11151/3542

## Data Availability

The data used to support the findings of this study are available from the corresponding author upon request.
